# Fever of unknown origin: 98 cases from Saudi Arabia

**DOI:** 10.4103/0256-4947.65259

**Published:** 2010

**Authors:** Mahmoud A. Moawad, Habib Bassil, Mona Elsherif, Abeer Ibrahim, Moustafa Elnaggar, Jameela Edathodu, Abdulaziz Alharthi, Muneerah Albugami, Ahmed Sabry, Mohammed Shoukri, Ibtisam Bakhsh, Ulrike Laudon

**Affiliations:** a From the Section of Internal Medicine, King Faisal Specialists Hospital and Research Centre, Riyadh, Saudi Arabia; b From the Section of Infectious Diseases, Department of Medicine, King Faisal Specialists Hospital and Research Centre, Riyadh, Saudi Arabia; c From the Department of Biostatistics, Epidemiology and Scientific Computing, King Faisal Specialists Hospital and Research Centre, Riyadh, Saudi Arabia

## Abstract

**BACKGROUND AND OBJECTIVES:**

Fever of unknown origin (FUO) is mainly secondary to infectious, neoplastic or inflammatory diseases. To increase the body of knowledge on this diagnosis in the region, we collected information on all patients admitted to our institution with FUO in a 13-year period.

**METHODS:**

We conducted a retrospective chart review of all immunocompetent males and females aged 13 years and older admitted between January 1995 and June 2008 who fulfilled the criteria for FUO. Data collection included demographics, laboratory investigations, imaging studies, procedures and discharge diagnoses. For true FUO, we recorded the duration of follow-up and the outcome.

**RESULTS:**

The 98 patients who met the criteria included 44 males and 54 females with a mean (SD) age of 41.3 (18.5) years and range of 14 to 85 years. The most frequent diagnostic etiology was infectious in 32 (32.7%). Seventeen (17.3%) patients were undiagnosed or had true FUO. Of 9 patients followed up, 8 recovered and 1 expired. The mean duration of follow-up was 20.6 months (range, 0-168 months).

**CONCLUSION:**

Infectious diseases, especially TB, continue to be the leading etiology of FUO in our area. Our data did not identify any predictor of certain FUO diagnoses except for older age and neoplastic etiology. True FUO patients generally did well. Reporting local experience is important in guiding clinicians about the epidemiologic patterns of FUO in their regions.

Fever of unknown origin (FUO) is defined as a temperature higher than 38.3°C on several occasions and lasting longer than 3 weeks, with a diagnosis that remains uncertain after 1 week of investigation in a hospital or outpatient setting.^1^,^2^ The condition is a diagnostic challenge and as such constitutes a significant number of referrals to tertiary care centers. Previous studies have described the spectrum of the disease to be mainly secondary to infectious, neoplastic or inflammatory diseases.^1^,^3^–^6^ Occasionally, miscellaneous diseases such as deep vein thrombosis (DVT) can present as FUO.^7^ Some of these cases, the incidence of which has varied between 9% and 30% in different studies, end without a diagnosis despite exhaustive workup.^3^–^9^ The prognosis of these patients, who are considered to have “true” FUO, was found to be generally good.^9^,^10^

The diagnostic workup for FUO is well described,^3^–^21^ but there is no “gold standard” and it may done differently depending on the clinical situation. In most cases, it begins by confirming the presence of fever in-hospital. A minimal workup includes a complete history and physical examination, including drug history, complete blood count with differential (CBCD), blood film, routine blood chemistry, urinalysis and microscopy, blood and urine cultures, anti-nuclear antibodies (ANA), rheumatoid factor (RF), HIV antibodies, cytomegalovirus (CMV), IgM antibodies, heterophile antibody test (if consistent with mononeucleosis-like syndrome), Q fever serology (if exposure to risk factors exists), chest X-ray and hepatitis serology (if liver enzymes are elevated).^11^ The role of different nuclear medicine studies (e.g., labeled leukocytes, gallium, indium and technetium scans) was emphasized in these patients, especially in ruling out inflammatory conditions.^14^–^21^

The published information about FUO in the Middle East is limited to studies from Turkey^4^,^5^,^22^–^23^ and Saudi Arabia.^6^,^24^ Our report should enrich the current knowledge on workup and etiology of FUO in the setting of a tertiary care center in the Middle East. Our objective was to describe the diagnostic workup, discharge diagnosis (or final diagnosis) of all patients admitted to our institution with FUO in a 13-year period. For those without a diagnosis after admission, i.e., patients with true FUO, we collected information on the duration of their follow-up and the outcome (recovery, persistence, mortality, establishment of diagnosis).

## METHODS

We conducted a retrospective chart review of all patients admitted to King Faisal Specialist Hospital and Research Centre (KFSHRC), Riyadh, Saudi Arabia, between January 1995 and June 2008 who were diagnosed as having FUO in their admission and/or discharge diagnoses. Inclusion criteria were immunocompetence and age 14 years or older, for all patients who fulfilled the criteria for FUO.^1^,^2^ We excluded patients who were already diagnosed with HIV infection, collagen vascular disease or malignancy. The data collection sheet included patient demographics (age, sex and nationality), admitting department, duration of fever before hospitalization, duration from hospitalization until diagnosis, number of admissions for FUO both locally and at KFSHRC, number of consultations and the type of consultation. Laboratory investigations included white blood cell counts (<4000, 4000-11 000 and ≥11 000), hemoglobin (≤100 g/L), aspartate aminotransferase (AST) (≤45 U/L), alanine aminotransferase (ALT) (≤45 U/L), bilirubin (≤21 μmol/L), lactate dehydrogenase (LDH), erythrocyte sedimentation rate (ESR), C-reactive protein (CRP), Brucella titer, rheumatoid factor (RF), anti-nuclear antibodies (ANA), thyroid-stimulating hormone (TSH), blood cultures, peripheral blood film for malaria, blood culture for fastidious organisms, urine culture, stool culture, sputum AFB-stain and culture, viral studies (HIV, HBV, HCV, CMV-IgM), and the tuberculin skin test. Imaging studies were chest X-ray, abdominal X-ray, bone X-ray, abdominal ultrasound (US), pelvis US, chest/abdomen/pelvis CT, chest/abdomen/pelvis MRI, nuclear medicine studies (indium, technetium, gallium, WBC-tagged), PET scan and lower extremity Doppler ultrasound (US). Data were also collected by other procedures such as echocardiography, upper endoscopy, colonoscopy, sigmoidoscopy, bronchoscopy, laparotomy, laparoscopy, bone marrow aspirate/biopsy, fine needle aspiration, tissue biopsy, fluid aspirate, and lumbar puncture. The number of days until final diagnosis was also recorded. For the non-diagnosed group, or the true FUO group, data collected included follow-up duration and outcome (mortality, recovery, establishment of diagnosis, and lost to follow-up.

The Chi-square test of significance was used to assess the correlation between pairs of categorical variables. The Mann-Whitney test was used to assess the significance of the difference between the medians of noncategorical continuous variables. A P value less than the set type I error rate of 5% was deemed significant. SAS software (version 9.1.3, www.sas.com) was used for computations and statistical analyses. The study was approved by the Clinical Research Committee and Research Ethics Committee of the KFSHRC Research Advisory Council (RAC # 2061 009).

## RESULTS

**Figure 1:**
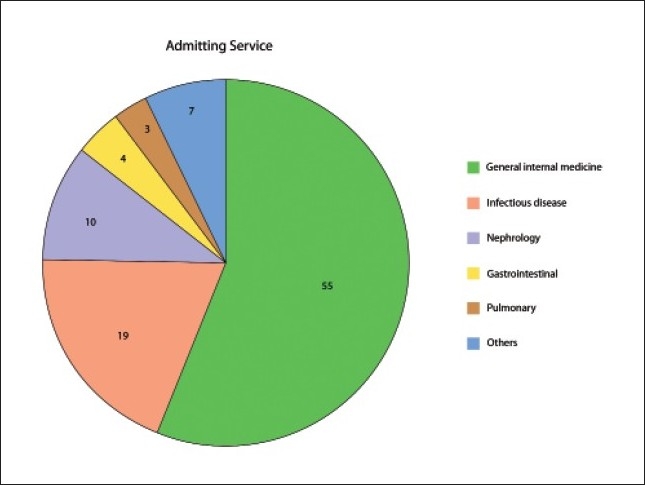
Number of patients admitted to each service (n=98).

Of 156 charts eligible for the study, 58 were either excluded or did not meet the inclusion criteria. The majority of those patients were labeled incorrectly as having FUO or pyrexia of unknown origin, with diagnoses being established in less than one week after admission; others were already diagnosed with cancer or HIV prior to their index admission, while a few were younger than 14 years of age. Of the remaining 98 patients, 44 were males and 54 were females. The mean age (SD) of the group was 41.33 years (18.56), and the range was 14 to 85 years. The majority of patients were admitted to the general internal medicine service, followed by infection diseases service (Figure 1). The mean (SD) duration of fever before hospitalization was 158 days (375); the median was 60 days, with a range of 21 to 2520 days. The mean (SD) number of admissions at local hospitals was 1.18 (1.00); and 1.27 (0.93) at KFSHRC. The mean number of consultations was 2.20 (1.43), with a range of 0.00 to 9.00. Ninety-four percent of patients needed consultation. Of those who needed consultations, 16% required consultations from the infectious disease service; 7%, from rheumatology service; 5%, from both; and 69%, from other services. Blood cultures were positive in 10 patients: coagulase negative staphylocci in 3, *S aureus* in 2, *Pseudomonas* in 2; and 1 positive set of each of the following: *Brucella, Salmonella typhi* and *Streptococcus pneumoniae*. Testing for HIV antibodies was done in 72 patients, and all were negative.

The most frequent imaging study done was chest X-ray, followed by CT chest/abdomen/pelvis, and then abdomen US and pelvis US. Only 2 patients did not get a chest X-ray; both were pregnant females in the first trimester. Almost half of the patients had an echocardiogram. The most frequent procedures carried out were bone marrow biopsy/ aspirate, tissue biopsy, FNA, fluid aspirate and bronchoscopy (Table 1). Splenectomy, kidney biopsy, liver biopsy and skin biopsy were performed for 1 patient each. The mean number of days to diagnosis was 53.54 days (152.24).

The most frequent diagnostic etiology was infectious [32 (32.7%) patients], of whom 19 (19.4%) had TB, followed by neoplastic disease [18 (18.3%)] and finally connective tissue diseases [14 (14.3%)]. Eighteen (18.3%) patients had miscellaneous diseases, and 17 (17.3%) patients were undiagnosed or were diagnosed to have true FUO (Table 2).

**Table 1:** Imaging and/or invasive procedures used in diagnosis of fever of unknown origin.

Imaging/procedure	No. (%)
Chest X-ray	96 (96.9)
CT chest, abdomen, pelvis	77 (78.5)
US abdomen	68 (69.4)
US pelvis	35 (35.7)
MRI chest, abdomen, pelvis	12 (12.2)
CT-PET whole body	19 (19.4)
Echocardiogram	50 (51.0)
Upper endoscopy	19 (19.4)
Colonoscopy	8 (8.2)
Sigmoidoscopy	3 (3.1)
Bronchoscopy	16 (16.3)
Laparascopy	5 (5.1)
BM aspirate/biopsy	58 (59.2)
FNA	34 (34.7)
Tissue biopsy	48 (49.0)
Fluid aspirate	23 (23.5)
Lumbar puncture	7 (7.1)

CT: computed tomography, US: ultrasound, MRI: magnetic resonance imaging, PET: positron emission tomography, BM: bone marrow, FNA: fine needle aspiration.

**Table 2:** Causes of FUO at KFSHRC 1995-2008 (n=98).

Diseases	No. (%)
Infectious diseases	32 (32.7)
Tuberculosis	19
Pulmonary	8
TB lymphadenitis	3
Extra-pulmonary, non-lymphadenitis	8
Other infections	13
Pyogenic	2
Typhoid	2
Rickettsial	2
Others	7
Connective tissue diseases	14 (14.3)
SLE	6
Adult Still disease	5
Rheumatoid arthritis	2
Behcet disease	1
Neoplasms	18 (18.3)
Hodgkin Disease	4
Non-Hodgkin lymphoma	7
Leukemia	1
Solid tumors	3
Others	3
Miscellaneous diseases	17 (17.3)
Fictitious	3
Ulcerative colitis	3
Venous thromboembolism	3
Drug-induced	2
Others	5
Undiagnosed (True FUO)	17 (17.3)

Among the 19 patients diagnosed with TB, 8 had pulmonary TB, diagnosed mainly through bronchoscopic bronchoalveolar lavage, pleural effusion cultures and mediastinal lymph node biopsy; 7 had extrapulmonary TB, mostly diagnosed through bone marrow biopsy and aspirate; 3 had TB lymphadenitis; 2 responded to empiric anti-TB therapy, 1 of whom for presumed TB-meningitis. The remaining 13 patients included 2 patients with *Salmonella typhi* and 2 patients with presumed rickettsial disease, based on a negative workup and dramatic response to empiric doxycycline treatment. Each of the other 9 patients had one of the following: *Klebsiella* liver abscess, *H influenzae* chronic pneumonia, chronic maxillary sinusitis, chronic active hepatitis B and C, CMV-urinary tract infection in a kidney transplant patient, *Salmonella* osteomyelitis in a patient with sickle cell disease, *Brucella, Shigella*, and streptococcal endocarditis.

The vast majority (15 of 18) of neoplastic cases were nonsolid tumors: Hodgkin lymphoma (4), non-Hodgkin lymphoma (7), Castleman disease (2), leukemia (1) and myelodysplastic syndrome (1). Only 3 cases were due to solid tumors: papillary thyroid cancer, colon cancer and metastatic adenocarcinoma of unknown primary origin.

The most common collagen-vascular diseases were systemic lupus erythematosus, followed by adult Still disease and rheumatoid arthritis. As a group, miscellaneous diseases were the third most frequent diagnosis after infectious and neoplastic diseases. This group included 16 patients: fictitious fever (3), ulcerative colitis (3), venous thromboembolism (extensive deep vein thrombosis, pulmonary edema and Budd-Chuari syndrome) (3), drug-induced (methyldopa, interferon) (2) and 1 case each of Sweet syndrome, sarcoidosis, cyclic neutropenia, usual interstitial pneumonitis with bronchiectasis; and bronchiolitis obilterans organizing pneumonia (BOOP).

Seventeen patients remained undiagnosed despite extensive workup; however, 8 were lost to follow-up. One patient left against medical advice after 23 days into his initial admission; another patient was discharged without diagnosis, for follow-up, but she never showed up. The other 6 patients were lost to follow-up at variable durations, between 1 and 12 months. One patient expired with multi-organ failure in the ICU during the initial admission. Out of the 8 patients who were discharged that had true FUO; 6 recovered without recurrence of their fever, another 1 continued to have recurrent low-grade fever without other symptoms, and the last patient expired after admission to the ICU with multi-organ failure 7 months later. The mean duration of follow-up was 20.6 months (0-168 months) (Table 2).

## DISCUSSION

FUO is a disease of young patients with an average age of 40 years. Our study confirmed the findings of previous studies—that infectious diseases remain the most frequently encountered cause of FUO, and that most of these cases are attributable to TB. Almost 1 out of 3 cases of FUO were secondary to an infectious etiology, and 1 out of 5 was secondary to TB. The neoplastic etiology was the next most frequent etiology, with a total of 18 patients. Connective tissue diseases were the final diagnoses in 14 patients; miscellaneous disease, in 17 patients; and 17 patients were diagnosed with true FUO.

The only reported cases of FUO from the Middle East were case series from Turkey and Saudi Arabia. In a pooled analysis of 857 patients with FUO from Turkey obtained from 13 articles, infections were the most common at 47.0%, of which 36.4% were secondary to TB; collagen vascular diseases were at 15.9%; neoplasms, 14.7%; and true FUO, 16.1%. The most common infections were TB and brucellosis; while the most common collagen vascular diseases were adult Still disease and SLE, and the most common neoplasms were Hodgkin and non-Hodgkin lymphoma. The authors looked at the cases reported in Turkey before 1984, in the period 1984 to 2002, and in 2004. They noted a trend toward decreased proportion, yet still the commonest diagnoses, of infectious diseases and TB etiology, and an increase in the proportion of noninfectious inflammatory diseases and undiagnosed cases or true FUO.^23^ Prior to our report, two studies were previously reported from Saudi Arabia. The first study was prospective and reported 62 cases in 1990.^6^ Comparing this report to ours, we noted a similar trend. The infectious etiology was found in about 57%, with TB accounting for 29%, connective tissue disease for 7%, neoplastic 13%, miscellaneous 11%; and true FUO for 11%. Comparing these results with our report shows that infectious disease, despite continuing to be the leading diagnosis, is less predominant than before (32% vs. 57%). This drop in infectious disease etiology was associated with an increase in connective tissue diseases, neoplastic diseases, miscellaneous diseases and true FUO (Table 3). The second report, from Saudi Arabia, was retrospective and included only 20 patients, a number that was too small to draw any conclusions.^7^ We reviewed the largest three Turkish series and pooled them with the three Saudi series (Table 3).

**Table 3:** Regional causes of fever of unknown origin in Turkey (TR) and Saudi Arabia (SA).

Study, year, country	Type	No. (%)	Mean age	Infection n (%)	TB n (%)	CTD n (%)	Neoplastic n (%)	Others n (%)	True FUO n (%)
Al-Mofleh 1990 (SA)^6^	P	62 (100%)	39.3	35 (56.5)	10 (28.6)	4 (6.5)	8 (12.9)	7 (11.3)	7 (11.3)
Tabak 2003 (TR)^4^	R	117 (100%)	35	40 (34.2)	28 (70.0)	27 (23.1)	19 (16.2)	12 (10.3)	16 (13.7)
Alaithan 2005 (SA)^24^	R	20 (100%)	41	7 (35.0)	3 (42.9)	2 (10.0)	3 (15.0)	5 (25.0)	3 (15.0)
Colpan 2007 (TR)^5^	P	71 (100%)	41.5	32 (45.1)	13 (40.6)	19 (26.8)	10 (14.1)	4 (5.6)	6 (8.5)
Kucukardali 2008 (TR)^23^	P	154 (100%)	42	53 (34.4)	21 (39.6)	47 (30.5)	22 (14.3)	8 (5.2)	24 (15.6)
Moawad 2008 (SA)	R	98 (100%)	41.3	32 (32.6)	19 (59.4)	14 (14.3)	18 (18.4)	17 (17.3)	17 (17.3)

**Total (%)**		**542 (100%)**	**40.0**	**199 (36.7)**	**95 (47.7)**	**113 (20.8)**	**80 (14.8)**	**54 (10.0)**	**74 (13.7)**

P: prospective, R: retrospective, TB: tuberculosis, CTD: connective tissue disease

The largest two European reports of FUO included 164 Romanian patients in 2003^3^ and 73 patients in the Netherlands in 2007.^25^ Infectious disease etiology was found in 45.1% of the patients in the former, compared to 16% in the latter; noninfectious inflammatory disease was comparable, at 18% vs. 22%; neoplasm was 25% vs. 7%; miscellaneous diseases, 2.1% vs. 4%. The incidence of reported nondiagnosed cases in the Romanian study was 7.3% as compared to 51% in the more recent report from the Netherlands.

Our study limitations include the retrospective design and the fact that it covered a span of 13 years, so that the results might reflect changes in the spectrum and standards of practice. Also, a large percentage of true FUO patients may have been lost to follow-up.

FUO continues to be a diagnostic challenge. Reporting local experience is important in guiding clinicians about the epidemiologic pattern in different regions. We have reported the largest case series of FUO from a tertiary care hospital in Saudi Arabia. The etiology of FUO was infection in roughly 1 out of 3 patients, and it was TB in 1 out of 5 patients. Pooling the results from the largest three Turkish series indicated that the infectious etiology was slightly more common (37%) and about half of those were TB. Our data did not identify any predictor of a certain FUO diagnosis except for older age and neoplastic etiology. About half of true FUO-diagnosed patients were lost to follow-up. Because of the large percentage of patients lost to follow-up from the true FUO group, it was difficult to draw any conclusion about their prognosis.

Our study showed there was a long duration of FUO before referral to our center. We believe that patients who fulfill the criteria of FUO should be referred early (within 1-4 weeks) to a tertiary care center where a multi-disciplinary approach and advanced diagnostic modalities are available. Many patients needed advanced nuclear medicine testing and PET scanning to secure the diagnosis. Up to 2002, most patients with FUO were admitted to the infectious diseases service, but for the last 5 to 6 years, the majority were admitted to the general internal medicine section, which is a reflection of the diversified etiologies.

TB continues to be the most frequent single diagnosis of FUO in our series, with less than half having pulmonary TB. For those patients, the diagnoses were confirmed by bronchoscopy and BAL. Our attempt to follow up patients with true FUO has highlighted a major problem of the health care delivery system in Saudi Arabia. The availability of a very high level of tertiary care was not coupled with a comparatively solid primary and secondary care system. Patients referred to our center, once diagnosed, were referred back to their “referring physician” or “referring hospital,” while others would simply select to be followed up in another tertiary care hospital(s). Fragmentation of care was obvious since more than half of our true FUO patients were lost to follow-up, and continuous, coordinated care was a rarity. The majority, if not all, may have sought care somewhere else. Coordination of care and building a strong primary- and secondary-care health care facilities are badly needed, and a national insurance program with a unique medical record number should facilitate coordination among different health institutions to deliver more efficient and effective care.
